# Numerical Simulation on Hydromechanical Coupling in Porous Media Adopting Three-Dimensional Pore-Scale Model

**DOI:** 10.1155/2014/140206

**Published:** 2014-04-17

**Authors:** Jianjun Liu, Rui Song, Mengmeng Cui

**Affiliations:** ^1^State Key Laboratory of Oil and Gas Reservoir Geology and Exploitation, Southwest Petroleum University, Chengdu 610500, China; ^2^School of Civil Engineering and Architecture, Southwest Petroleum University, Chengdu 610500, China; ^3^School of Petroleum and Natural Gas Engineering, Southwest Petroleum University, Chengdu 610500, China

## Abstract

A novel approach of simulating hydromechanical coupling in pore-scale models of porous media is presented in this paper. Parameters of the sandstone samples, such as the stress-strain curve, Poisson's ratio, and permeability under different pore pressure and confining pressure, are tested in laboratory scale. The micro-CT scanner is employed to scan the samples for three-dimensional images, as input to construct the model. Accordingly, four physical models possessing the same pore and rock matrix characteristics as the natural sandstones are developed. Based on the micro-CT images, the three-dimensional finite element models of both rock matrix and pore space are established by MIMICS and ICEM software platform. Navier-Stokes equation and elastic constitutive equation are used as the mathematical model for simulation. A hydromechanical coupling analysis in pore-scale finite element model of porous media is simulated by ANSYS and CFX software. Hereby, permeability of sandstone samples under different pore pressure and confining pressure has been predicted. The simulation results agree well with the benchmark data. Through reproducing its stress state underground, the prediction accuracy of the porous rock permeability in pore-scale simulation is promoted. Consequently, the effects of pore pressure and confining pressure on permeability are revealed from the microscopic view.

## 1. Introduction

Above all fluid transport properties of porous media at in situ conditions, permeability has been conducted as a critical topic in many research fields such as oil and gas production, carbon dioxide storage, underground gas storage, coal mining, and fuel cell [1–4]. Wide ranges of stress dependent permeability tests application in porous media have led to myriad experimental research [5–8]. However, it is difficult to investigate the inner process of the rock matrix deformation and the fluid flow. Besides, there are an infinite number of possible fluid arrangements in terms of saturations and displacement series to constitute a comprehensive set of experimental measurements. The almost universal practice in the oil industry is to measure the permeability of certain core samples at one initial condition and then use empirical models, of limited accuracy, to predict the whole behavior for in situ cases [9].

In recent years, pore-scale modeling representing microstructures of porous media has been widely used for fluid transport properties prediction. Breakthrough in this field can be summarized as two aspects: lattice Boltzmann method (LBM) and finite element method (FEM). In LBM, the discrete form of Boltzmann equation, which is solved over a regular lattice grid, is used to conduct the simulation of fluid flow in the porous media [10, 11]. While Navier-Stokes equations are usually adopted in FEM [12], two-dimensional (2D) [13, 14] and three-dimensional (3D) [15–17] pore networks of heterogeneous porous media are used in these simulations. The 2D models usually reproduce disorder system in porous media properly but are unable to reproduce the spatial interconnectivity of pore systems. And in most 3D models, different sizes of spheres and cylinders serve as pores and throats of the porous stone. GAO has made breakthroughs in reconstructing the 3D real shape porous model based on micro-CT images using the lattice Boltzmann method [18]. Yet, the hydromechanical coupling at in situ condition has not been taken into consideration in the LBM simulations.

This paper proposes a novel approach of reconstructing the 3D finite elements model from natural porous media images employing the commercial software MIMICS [19] and ICEM [20], which are prior to the pore network model based on some basic assumptions. These 3D FEM models are able to reproduce the real shape of natural porous media compared to most LBM models. And the FEM-based software ANSYS [20] and CFX [20] can realize the two-way hydromechanical coupling through workbench platform. The tetra finite volume elements of different sizes are used to represent the origin microstructures of porous media rather than some regular shapes (sphere or cylinder, etc.). Parameters of the sandstone samples, such as the stress-strain curve, Poisson's ratio, and permeability under different pore pressure and confining pressure, are tested in laboratory scale. Four physical models possessing the same pore and rock matrix characteristics as the natural sandstones have been developed. Hereby, permeability of four samples under different pore pressure and confining pressure has been predicted. The results between the numerical simulation and experiment are compared, accordingly.

## 2. Sample Preparation

**Figure 1:**
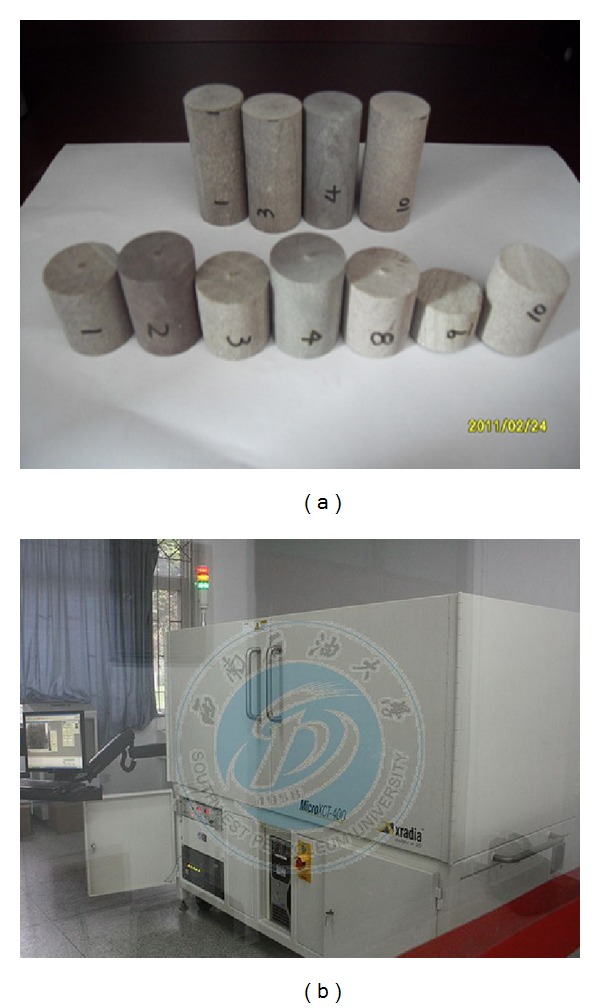
Sandstone samples and equipment used in this research. (a) is initial sandstone samples; (b) is Micro X CT-400.

**Figure 2:**
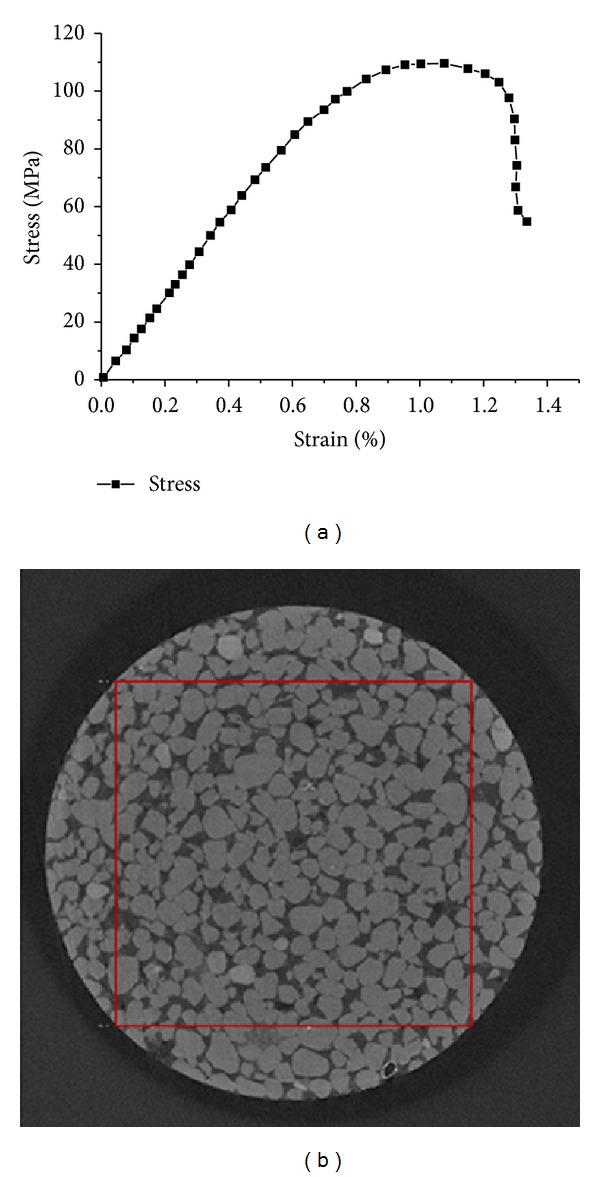
Basic data for rock mechanics experiment and CT imaging. (a) is stress-strain curve; (b) is cross-section image of sandstone sample S1.

Sandstone core samples used in this paper are provided by the Daqing Oilfield in China. As is shown in Figure 1(a), sandstone samples taken from adjacent parts of the same block of rock are used as input for the rock properties test including Young's modulus, Poisson's ratio, permeability, and porosity. All of these parameters are measured by standardized equipment in laboratory. Micro-CT scanner (Figure 1(b)) is employed to image the samples. Stress-strain curve of the sandstone sample, which is used as material property for the structural analysis in the simulation, is shown in Figure 2(a).

In the imaging process, small cylinders are drilled out of larger core samples for scanning. Then, the pore space is evacuated to inject a mixed resin of Epoxy resin and Struers Caldofix, which is typical for embedding of materialographic specimens. The resin is impregnated into the samples to maintain the integrity of sandstones during the physically destructive drilling to obtain small size samples. The small samples are imaged by the Micro X CT scanner in the State Key Laboratory of Oil and Gas Reservoir Geology and Exploitation in Southwest Petroleum University. One cross-section of micro-CT images is shown in Figure 2(b). A total of four sandstone images (numbered S1, S2, S3, and S4) are used in this paper. And a cube of voxels is extracted from the origin micro-CT images. For example, 400 × 400 × 400 pixels of S1 are selected and used to construct the pore-scale model, which is marked by the red block in Figure 2(b).

## 3. Finite Elements Model for Rock Matrix and Pore Space

**Figure 3:**
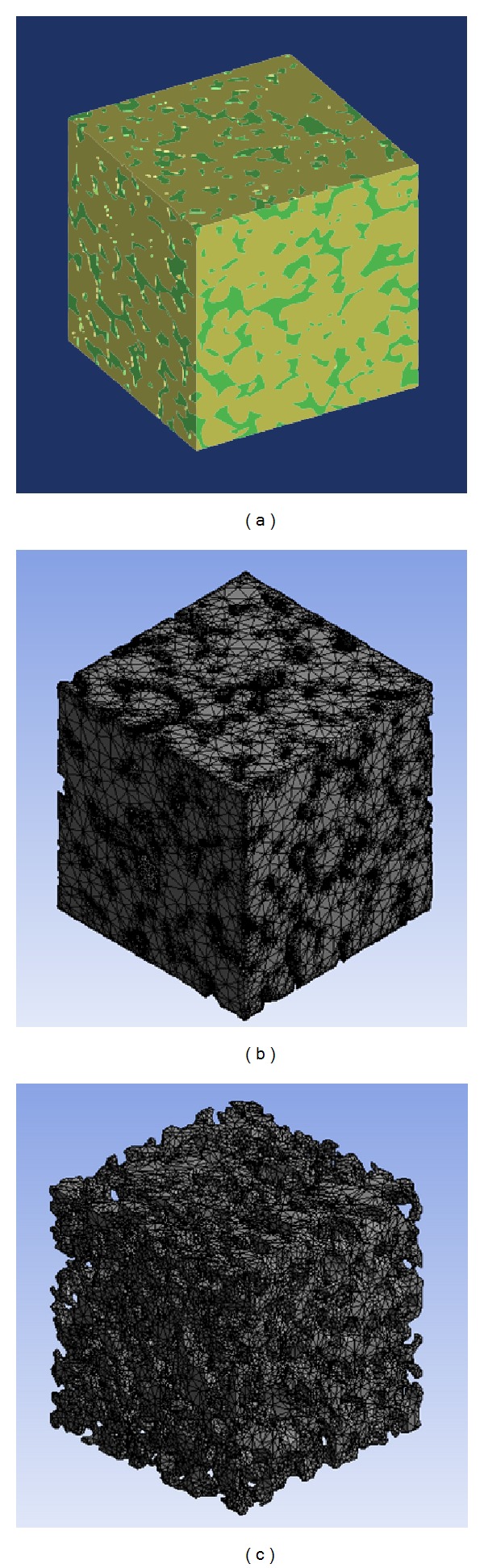
Reconstruction model, matrix, and pore space finite element model of S2.

**Figure 4:**
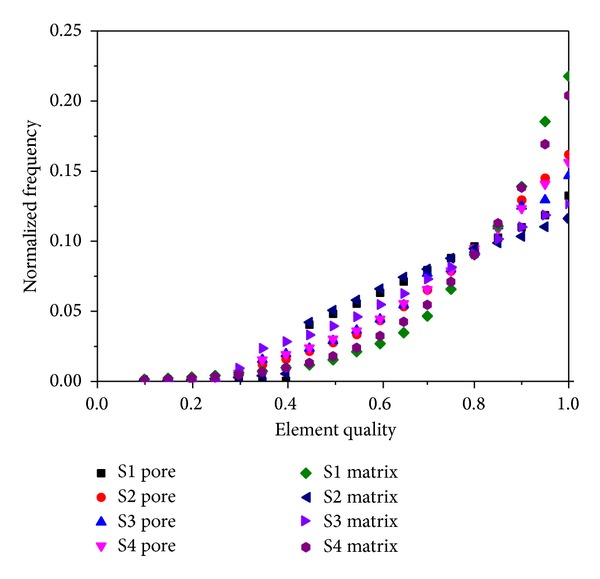
Element quality of reconstructed FEM models.

A challenging problem in numerical simulation of fluid properties in porous medium is how to build mesh models for pore space, as well as for the rock matrix. Moreover, both of the models must match each other perfectly to ensure the accuracy of hydromechanical simulation. Here, MIMICS software is employed to establish the 3D finite elements models based on the micro-CT images. The rock matrix and pore space are distinguished by the threshold of different color, after importing the micro-CT image data with a  .*raw *suffix to the MIMICS software. Then surface meshes are transformed into tetrahedral elements wrapping the solid and the pore models. Based on surface meshes, mapping algorithm is adopted to generate 4-node tetrahedral element meshes. To ensure the accuracy of the simulation, the rock matrix and pore boundaries must be fitted to each other. Thus, nonmanifold assembly function in MIMICS is used when generating the volume meshes, which means the volume meshes of rock matrix and pore space are generated as assembly. The reconstructed model as well as the finite element models of S2 is shown in Figure 3. Meanwhile, the boundary meshes are selected to form the different parts in ICEM software, making preparations for the application of the boundary conditions into the numerical simulation. And basic parameters of the models used in this study are listed in Table 1. The element quality of both pore space and rock matrix models is shown in Figure 4.

## 4. Mathematical Model

The continuity equation represents conservation of mass for fluid flow, which can be written as [14],

(1) $$\begin{array}{c} \Delta \cdot v=0. \end{array}$$

Here *v* represents the velocity of fluid flow. The fluid is assumed to be continuous and incompressible.

Navier-Stokes equation, representing conservation of momentum for the fluid flow, can be written as [14],

(2) $$\begin{array}{c} \rho \left[\frac{\partial v}{\partial t}+\left(v\cdot \nabla \right)v\right]=-\nabla p_{p}+\rho g+\mu \left(\nabla v+\nabla^{T}\right). \end{array}$$

Here *v* is velocity of fluid flow, *ρ* is density of fluid, *μ* is dynamic viscosity of fluid, *p*_*p*_ is fluid pressure, and *g* is acceleration due to gravity. Fluid is assumed to be incompressible and Newtonian.

By the CFX software, the outlet flow rate *Q* can be acquired. Then the absolute permeability will be calculated in the following term:

(3) $$\begin{array}{c} K=\frac{\mu Q}{L\Delta p}. \end{array}$$

Here, *L* is the length of the cubic sandstone finite element model.

The pressure on the fluid-solid interface is transferred to each other by the following equation, which is called principle of effective stress firstly proposed by Terzaghi [21]:

(4) $$\begin{array}{c} f_{i}=p_{c}-\alpha p_{p}. \end{array}$$

Here *p*_*c*_ is the confining pressure, and *α* is the coefficient, ranging from 0 to 1. In the simulation, it is defined as 1.

The three-dimensional equilibrium differential equation is [22]

(5) $$\begin{array}{c} \underset{j}{\sum }\frac{\partial \sigma_{ij}}{\partial x_{j}}+f_{i}=0. \end{array}$$

Here *σ*_*ij*_ is the stress tensor, and *f*_*i*_ is the body force.

The three-dimensional geometric equations of the rock matrix are [22]

(6) εx=∂u∂x,  εy=∂v∂y,  εz=∂w∂z,γxy=∂v∂x+∂u∂y,  γyz=∂v∂z+∂u∂y,γzx=∂w∂x+∂u∂z.

Here *ε*_*x*_, *ε*_*y*_, and *ε*_*z*_ are the normal strain, *γ*_*xy*_, *γ*_*yz*_, and *γ*_*zx*_ are the shear strain, and *u*,*v*, and *w* are the displacement components.

The elastic physical equations are [20]

(7) $$\begin{array}{c} \varepsilon_{x}=\frac{1}{E}\left[\sigma_{x}-\mu \left(\sigma_{y}+\sigma_{z}\right)\right],\;\;\gamma_{xy}=\frac{2\left(1+\mu \right)}{E}\tau_{xy},\\ \varepsilon_{y}=\frac{1}{E}\left[\sigma_{y}-\mu \left(\sigma_{x}+\sigma_{z}\right)\right],\;\;\gamma_{yz}=\frac{2\left(1+\mu \right)}{E}\tau_{yz},\\ \varepsilon_{z}=\frac{1}{E}\left[\sigma_{z}-\mu \left(\sigma_{y}+\sigma_{x}\right)\right],\;\;\gamma_{zx}=\frac{2\left(1+\mu \right)}{E}\tau_{zx}. \end{array}$$

Here *E* is modulus of elasticity and *μ* is Poisson's ratio.

## 5. Fluid-Solid Coupling Simulation 

**Figure 5:**
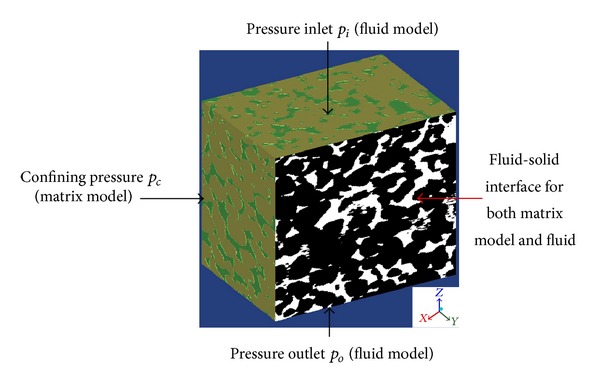
Boundary conditions in the simulation.

The structural analysis for matrix model and fluid flow for pore model are solved by ANSYS solver and CFX solver, respectively. As a result, boundary conditions of the matrix model and pore model should be set in ANSYS workbench separately. Firstly, confining pressure (*p*_*c*_) for structural analysis is added to the four sides of the cubic matrix models. The data of the stress-strain curve shown in Figure 2(a) is assigned as material parameters of rock matrix. Poisson's ratio of the origin sandstone sample tested by triaxial test is 0.341. And the contact surfaces of solid and fluid inside the rock are defined as fluid-solid interface, through which pressure is transmitted between the solid and fluid. Meanwhile, pressure inlet and outlet conditions are applied to the top and the bottom of the pore model along *z* direction. Water is used as the fluid medium in the simulation. Fluid field will be solved by CFX solver at the beginning of simulation. After the fluid pressure on the fluid-solid interface is transferred to ANSYS structural solver, the meshes on the fluid-solid interface of both rock matrix and pore model will be remeshed according to the deformation data. The iteration of the fluid field and deformation of rock matrix is continued until the models converge. The brief schematic of boundary conditions in the simulation is shown in Figure 5. A laminar flow is assumed. The pressure-corrections scheme SIMPLE is used for velocity-pressure coupling. All simulations are converged at different iterations under the condition that the absolute convergence criteria are set to 1E-5 for all equations, under the condition of which default relaxation factors are used.

**Figure 6:**
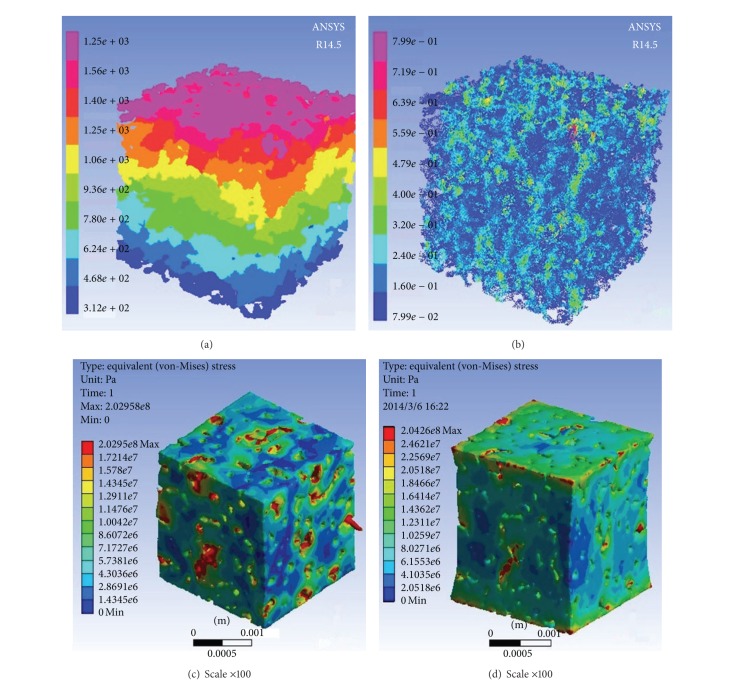
Simulation results images. (a) and (b) are the images of fluid pressure contours and velocity vectors at the pressure gradient 10 MPa/m (*p*_*i*_ = 1538 Pa, *p*_*o*_ = 0 Pa), (c) is the von-Mises stress field of S2 rock matrix at the pore pressure of 20 MPa (*p*_*i*_ = 2*e*7 + 1538 Pa, *p*_*o*_ = 0 Pa), and (d) is the von-Mises stress field of S2 rock matrix at the condition of *p*_*c*_ − *p*_*p*_ = 20 MPa.

**Figure 7:**
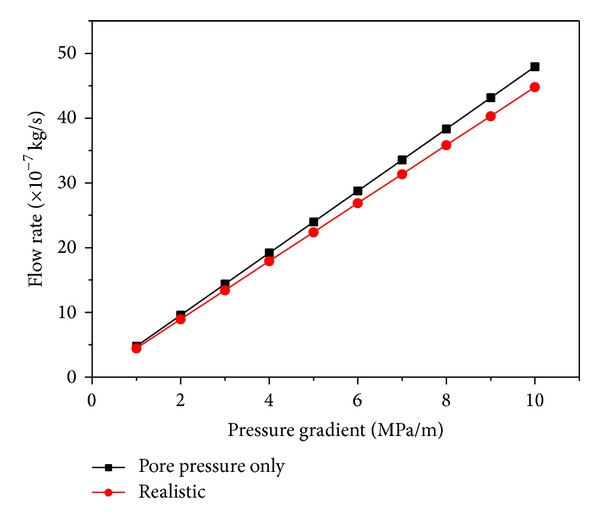
Flow rate of outlet versus fluid pressure gradient. The red curve is traditional simulation that does not consider the hydromechanical coupling; the black curve illustrates the state of hydromechanical coupling when the confining pressure is regarded as 0 and the pore pressure is 2 MPa.

The images of fluid pressure contours and velocity vectors along *z* direction are shown in Figures 6(a) and 6(b). It can be seen that fluid flow along some channels with good connectivity mainly. This phenomenon has been verified by classical microfluid experiment in porous media. As mentioned before, most simulation on seepage mechanism in pore-scale models of porous media has not taken hydromechanical coupling into consideration, which do not keep up with the actual condition. Due to the fact that the tested samples are cylindrical, the benchmark experimental data is merely along *z* direction. The same boundary conditions are adopted in the simulation. Here, pore pressure (*p*_*p*_ = 2 MPa) is adapted to simulation for its effect on the rock permeability.  Figure 7 shows that the seepage flow rate of the outlet is higher under fluid-solid coupling condition when taking the pore pressure into consideration than that in traditional simulation. Actually, the confining pressure is usually greater than the pore pressure to guarantee the accuracy in the experiments.

**Figure 8:**
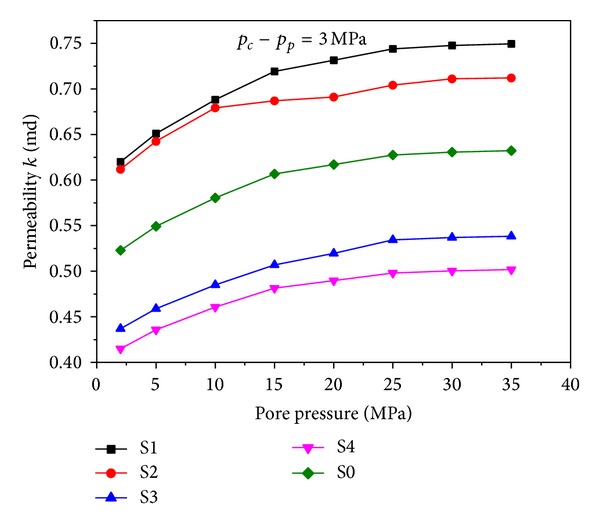
Permeability versus pore pressure at the condition of *p*_*c*_ − *p*_*p*_ = 3 MPa. S1, S2, S3, and S4 are simulation results; S0 is the experimental data.

In this paper, the same boundary conditions are adopted for both simulation and experiment. As is shown in Figure 8, curves of rock permeability versus pore pressure at the condition of *p*_*c*_ − *p*_*p*_ = 3 MPa are plotted. It is found that the rock permeability changes at the fixed effective pressure level. The rock permeability increases along with the increase of pore pressure at the same effective pressure initially and curves become relatively flatter when pore pressure exceeds 15~20 MPa. It is a common sense that permeability of porous media depends on the size and connectivity of throats (the narrow openings connecting the pores). In fact, when fluid is injected into the porous media, the pore space will be extendedly driven by the pore pressure. Moreover, stress concentration usually occurs because of the irregular matrix shape of the porous media. As is shown in Figure 6(c), large deformation occurs under the pore pressure at some weak zone, which eventually leads to the increase of rock permeability. This reveals that the phenomenon of how water flooding microscopically, the most widely used well stimulation in the development of oil field, promotes the permeability of oil reservoir.

**Figure 9:**
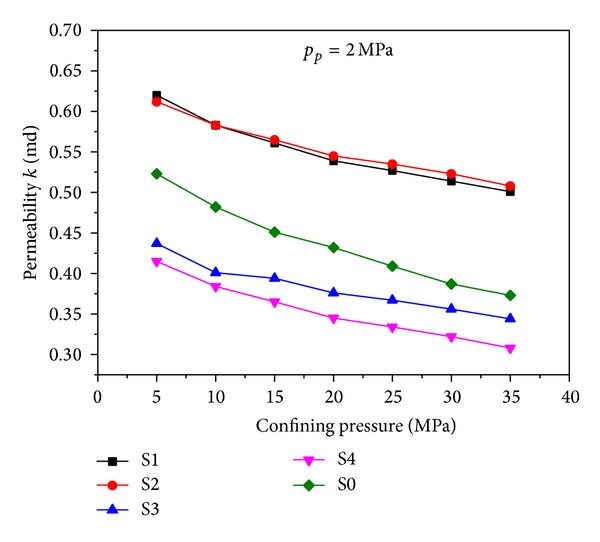
Permeability versus confining pressure at the condition of *p*_*p*_ = 2 MPa. S1, S2, S3, and S4 are simulation results; S0 is the experimental data.

Meanwhile, curves of rock permeability versus confining pressure at the condition of *p*_*p*_ = 2 MPa are plotted in Figure 9. The permeability decreases with the increase of confining pressure at the beginning and curves become less steep when confining pressure exceeds 20~25 MPa. Analogically, Figure 6(d) shows that when *p*_*c*_ − *p*_*p*_ = 20 MPa, the rock matrix is compressed and stress concentration occurs in the same parts as Figure 6(c). The trends of simulation result agree well with the experimental result. All of these verify the feasibility of the simulation and the necessity of conducting hydromechanical coupling analysis when predicting the fluid properties in porous media.

## 6. Conclusion

Based on micro-CT images scanned by the State Key Laboratory of Oil and Gas Reservoir Geology and Exploitation in the Southwest Petroleum University, four sandstone 3D finite element models are generated using MIMICS and ICEM software. Through the hydromechanical coupling simulation on fluid transport properties in pore-scale models of porous media by ANSYS and CFX software, permeability of sandstone samples under different pore pressure and confining pressure is predicted. Good agreements are acquired on simulation result against the benchmark data. This indicates that the hydromechanical coupling analysis is able to promote the accuracy of permeability prediction of rock through reproducing its stress state underground.

It should be noted that this study provides a preliminary study for microfluids in deformable porous media using finite element methods. Though examples used in this study mainly focus on the petroleum industry, it can also be widely applied to hydromechanical coupling analysis in other relevant area.

## Figures and Tables

**Table 1:** Parameters of sandstone samples used in this study.

Number	Resolution (*μ*m)	Size	Finite elements number		Porosity Φ
			Matrix	Pore	
Origin sample	—	*D* = 2.5 cm, *L* = 5 cm	—	—	15.34%
S1	3.845	400 × 400 × 400 pixels	1336224	230207	21.03%
S2	5.133	300 × 300 × 300 pixels	289757	222188	18.33%
S3	5.133	300 × 300 × 300 pixels	546717	365739	17.28%
S4	5.133	300 × 300 × 300 pixels	279167	376709	11.22%
